# Comprehensive analysis of autophagy-related genes and patterns of immune cell infiltration in valvular atrial fibrillation

**DOI:** 10.1186/s12872-021-01939-1

**Published:** 2021-03-11

**Authors:** Ao Liu, Kangni Jia, Huaibin Liang, Qi Jin

**Affiliations:** 1 Department of Cardiology, Shanghai Ruijin Hospital, Shanghai Jiao Tong University School of Medicine, No. 197, Shanghai Ruijin Er Road, Shanghai, 200025 China; 2 Department of Neurology, Shanghai Ruijin Hospital, Shanghai Jiao Tong University School of Medicine, Shanghai, China

**Keywords:** Valvular heart disease, Atrial fibrillation, Bioinformatics, Autophagy, Immune cell

## Abstract

**Background:**

The development of atrial fibrillation (AF) following valvular heart disease (VHD) remains a common disease and is associated with substantial adverse complications. However, valid molecular diagnostic and therapeutic tools for post-VHD AF have not been fully established. This study was conducted to discover the molecular mechanisms and immune microenvironment underlying AF following VHD.

**Methods:**

Gene expression profiles of the GSE41177 dataset were assessed to construct a protein–protein interaction network, and then, autophagy-related hub genes were identified. In addition, to determine the functions of immune cell infiltration in valvular AF, we used the CIBERSORT algorithm to estimate the composition of 22 immune cell types in valvular heart disease. Finally, correlation analysis was carried out to identify the relationship between differentially expressed autophagy-related genes (DEARGs) and significant immune cell subpopulations to reveal potential regulatory pathways.

**Results:**

A total of 153 DEARGs were identified in AF-VHD patients compared with controlled donors. Moreover, we screened the top ten hub nodes with the highest degrees through a network analysis. The ten hub nodes were considered hub genes related to AF genesis and progression. Then, we revealed six significant immune cell subpopulations through the CIBERSORT algorithm. Finally, correlation analysis was performed, and six DEARGs (BECN1, GAPDH, ATG7, MAPK3, BCL2L1, and MYC) and three immune cell subpopulations (T cells CD4 memory resting, T cells follicular helper, and neutrophils) were identified as the most significant potential regulators.

**Conclusion:**

The DEARGs and immune cells identified in our study may be critical in AF development following VHD and provide potential predictive markers and therapeutic targets for determining a treatment strategy for AF patients.

**Supplementary Information:**

The online version contains supplementary material available at 10.1186/s12872-021-01939-1.

## Introduction

Atrial fibrillation (AF), a surging global health care burden, affects nearly 1–4% of the adult population in the USA, and this number is expected to exceed 13% for individuals older than 80 years of age [1]. Valvular heart disease (VHD) is also a common disease worldwide [2]. The common causes of VHD are degeneration and some modifiable risk factors (such as elevated blood pressure and lipid profiles) in higher income countries, whereas rheumatic heart disease is a common cause in developing regions [3]. Importantly, AF patients with concomitant VHD are at higher risk of stroke and systemic embolism than those without VHD [4].

Autophagy is a self-digesting mechanism that maintains cellular homeostasis by eliminating unnecessary or dysfunctional cellular components [5, 6]. The Human Autophagy Database (HADb) is a web-based resource, that provides a comprehensive and up-to-date list of human genes and proteins involved in autophagy [7]. Previous studies have reported that autophagy is a potential novel mechanistic contributor to the pathological processes of AF genesis [8, 9]. Nakano et al. showed that AF patients displayed a significant decrease in the expression level of mitochondrial ALDH2, which regulates cardiac autophagy [10]. These findings suggest that autophagy and AF may have underlying relationships. Additionally, immune cell infiltration in the atrial myocardium is common in patients with either lone AF or valvular AF [11, 12]. A previous study observed that activated T lymphocytes (CD3^+^ and HLA-DR^+^) were significantly up-regulated in the peripheral blood of AF patients compared with individuals with sinus rhythm (SR) (36% vs 27%; *P* < 0.001) [13]. Moreover, this up-regulation was reversed when SR was maintained after cardioversion at follow-up [13]. These findings suggested that immune infiltration may play an essential role in AF development. In addition, cross talk between components of autophagy and immunity has also been reported; regulators of autophagy control regulators of inflammation, and vice-versa [14]. However, the communicative regulatory mechanisms of autophagy and immunity in the initiation and maintenance of AF remain unknown.

In this study, a protein–protein interaction (PPI) network of differentially expressed autophagy-related genes (DEARGs) was constructed, and hub genes were revealed. To determine the functions of DEARGs in valvular AF, we constructed a valvular AF-related TF/mRNA/miRNA network by integrating all TF-DEARG and miRNA-DEARG interactions. In addition, we used the CIBERSORT algorithm to estimate fractions of the immune cell subpopulations in VHD samples [15]. Finally, we conducted co-expression analysis with DEARGs and immune cells to identify the underlying regulatory mechanisms in AF genesis.

## Materials and methods

Supplementary methods are available in Additional file 1.

### Differentially expressed mRNA microarray datasets and data processing

The human VHD gene expression profile GSE41177 dataset [16] was downloaded from the Gene Expression Omnibus [17] database. The mRNA expression data in the GSE41177 dataset were obtained from 16 persistent AF patients and 3 patients with normal SR undergoing valvular surgery. Gene expression values of |log2 fold-change (FC)|> 1 and adjusted original *P*-values (adj. *P*-values) < 0.05 were used to identify differentially expressed mRNAs (DEmRNAs) in the AF samples.

### Identification of differentially expressed autophagy-related genes (DEARGs)

We extracted 232 human autophagy-related genes from the HADb. Then, we obtained the DEARGs by intersecting the 232 ARGs with DEmRNAs identified in the GSE41177 dataset.

### GO and pathway enrichment analyses of the DEARGs

Gene Ontology (GO) and Kyoto Encyclopedia of Genes and Genomes (KEGG) pathway enrichment analyses of the DEARGs were performed using Database for Annotation, Visualization and Integrated Discovery (DAVID 6.8) [18]. KEGG pathway enrichment analysis for these DEARGs was carried out to reveal the autophagy gene-associated pathways.

### Construction and analysis of the PPI network of DEARGs

The Search Tool for the Retrieval of Interacting Genes (STRING database, V11.0) was used to predict the interactions of these DEARGs and create a PPI network [19]. Subsequently, after downloading STRING database results with a confidence score > 0.7, the biological networks and topological features were visualized and analyzed using Cytoscape software [20]. Then, hub genes were revealed using CytoHubba, a plugin of Cytoscape.

### Prediction of miRNAs and transcription factors (TFs) that regulate DEARGs

In this study, TF-DEARG interactions were predicted by two different TF-target prediction algorithms in the Enrichr database [21]: TRANSFAC and JASPAR. Next, we used miRTarBase in the Enrichr database to retrieve miRNA-mRNA interactions. The integrated TF/mRNA/miRNA regulatory network was constructed using Cytoscape based on the identified TF-DEARG and miRNA-DEARG interactions.

### CIBERSORT estimation

Cell Type Identification by Estimating Relative Subsets of RNA Transcripts (CIBERSORT) algorithm is a comprehensive analytical tool constructed by Newman et al. [15] to estimate the immune cell composition on the basis of gene expression profiles. In the present study, the fractions of 22 immune cell subpopulations in the SR-VHD and AF-VHD samples were estimated by CIBERSORT. Only cases with CIBERSORT output of *P* < 0.05 were included in further analysis. The Wilcoxon rank-sum test was performed to identify significant immune infiltration cell subpopulations between the SR-VHD and AF-VHD samples.

### Correlation analysis of the ARGs and immune cells in valvular AF

Ultimately, the Pearson correlation analysis was implemented to reveal the relationship between the DEARGs and immune cells. We analyzed the correlation of DEARG expression with significant immune cell subpopulations in the VHD samples.

## Results

### Flowchart of the analysis process

**Fig. 1:**
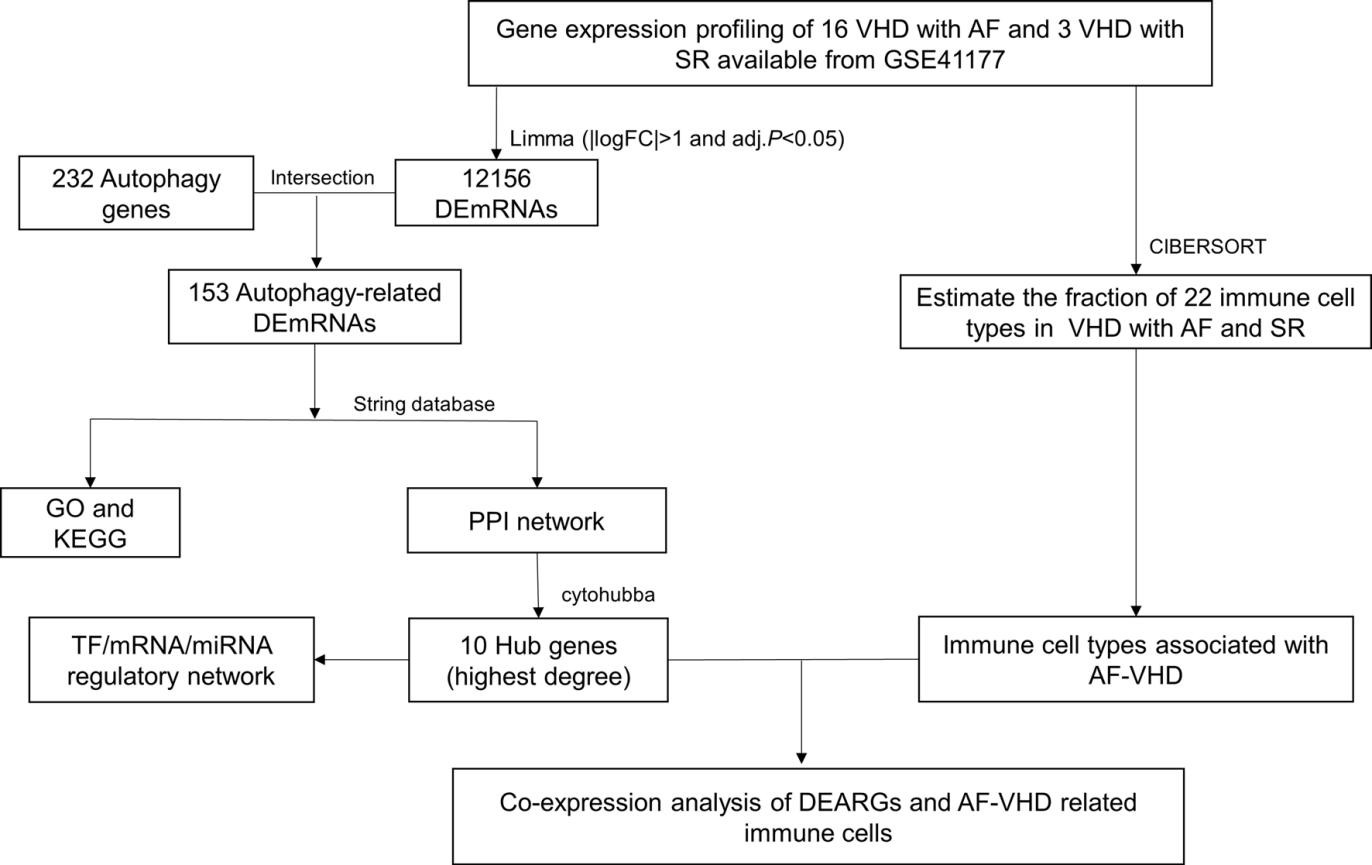
The flowchart of the analysis process

Figure 1 summarizes the analysis process of this study. First, we calculated DEmRNAs with |logFC| > 1 and adj. *P*-value < 0.05 in the GSE41177 dataset. We selected 153 DEARGs from HADb for further analysis. Second, we identified ten autophagy-related hub genes from the PPI network and constructed the autophagy-related TF/mRNA/miRNA regulatory network. Third, we revealed six differential immune cell subpopulations that reached the threshold criterion, *P*-value < 0.05, in the CIBERSORT algorithm. Finally, we conducted co-expression analysis of the DEARGs and differential immune cell subpopulations. In conclusion, we identified six DEARGs (BECN1, GAPDH, ATG7, MAPK3, BCL2L1, and MYC) and three immune cells (T cells CD4 memory resting, T cells follicular helper, and neutrophils), which may play crucial roles in the molecular mechanisms and immune microenvironment underlying valvular AF.

### Identification of DEARGs in valvular AF

**Fig. 2:**
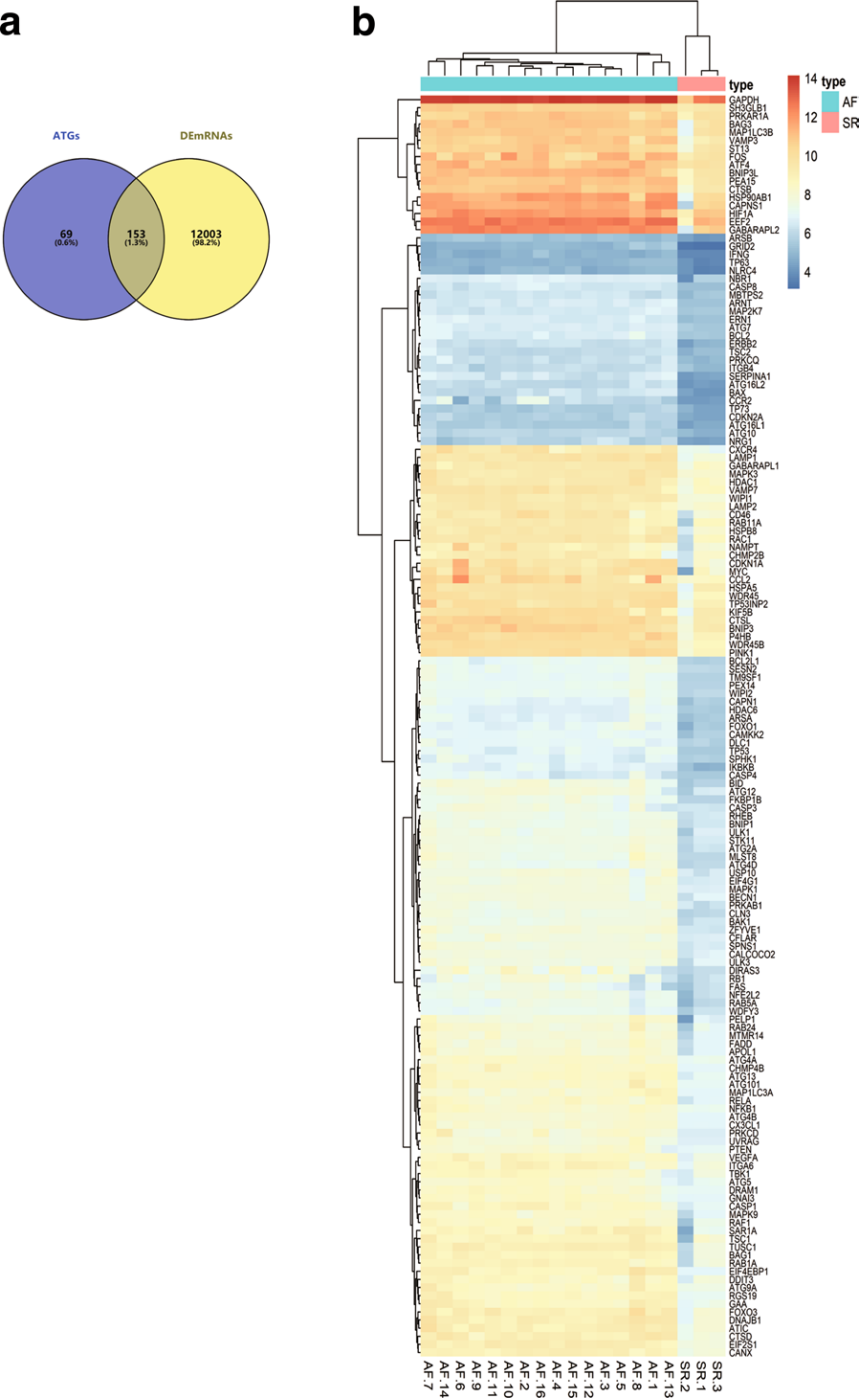
Hierarchical clustering analysis of differentially expressed autophagy-related genes (DEARGs). **a** Venn diagram of intersecting DEARGs. The dark area in the middle represents the ARGs that were identified through both analysis of differentially expressed mRNAs and the ARGs in the Human Autophagy Database (HADb). **b** Heatmaps of the DEARGs. The up-regulated DEARGs are marked in red, whereas the down-regulated DEARGs are marked in blue

We identified 12,156 mRNAs in 16 valvular AF samples and 3 SR tissues that exhibited significantly differential expression. Simultaneously, 232 autophagic genes were obtained from HADb. Then, the 232 autophagic genes were intersected with the 12,156 DEmRNAs identified in the GSE41177 dataset. The results showed that 153 DEARGs were suitable for further analysis (|logFC| > 1 and adj. *P*-value < 0.05; Fig. 2a). In addition, the gene expression of the 153 selected DEARGs is shown in heatmaps in Fig. 2b.

### GO and pathway enrichment analyses of the DEARGs

**Fig. 3:**
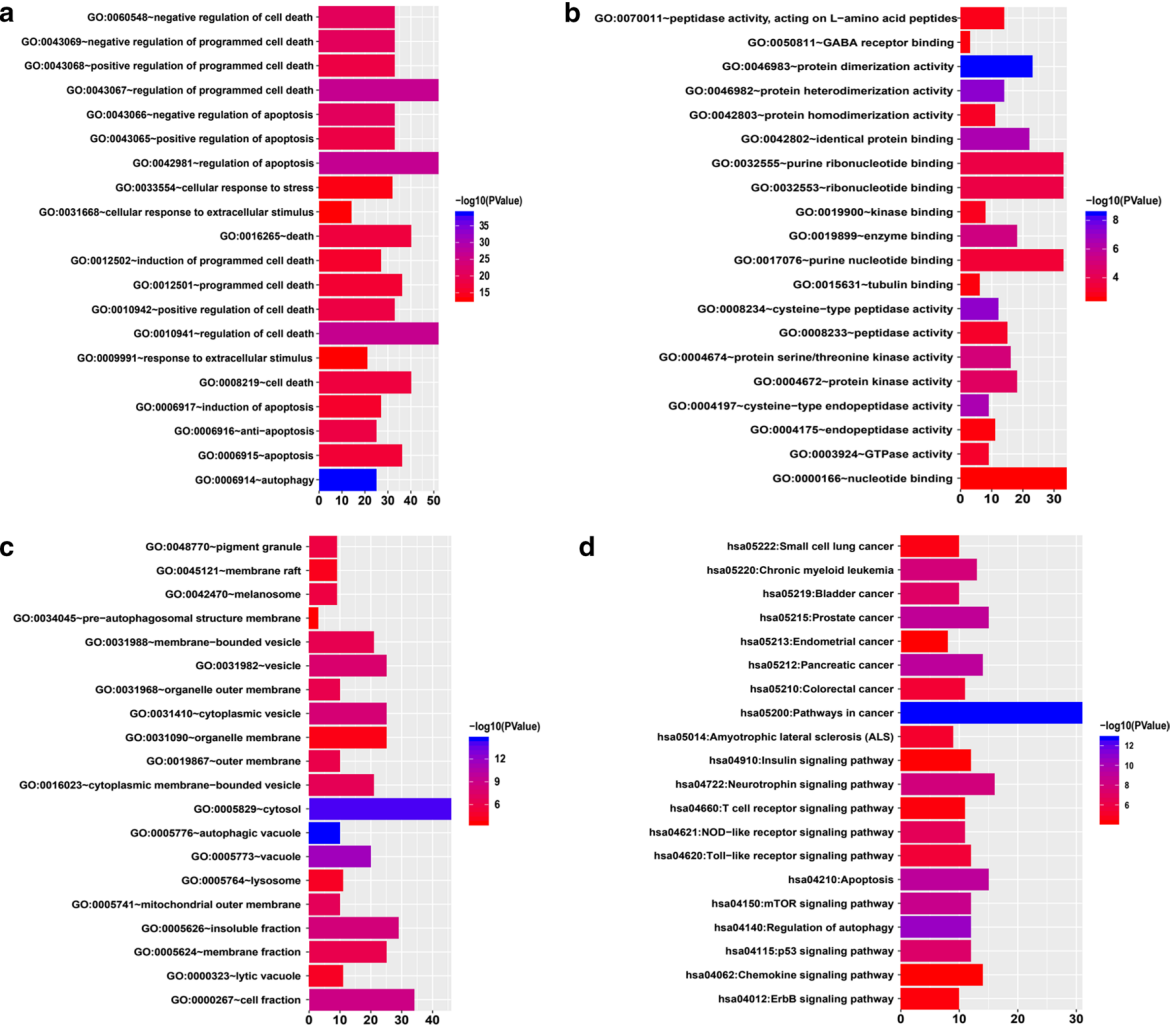
Gene Ontology (GO) and Kyoto Encyclopedia of Genes and Genomes (KEGG) pathway enrichment. **a**–**c** Bar plot of significant GO terms showing DEARG enrichment in biological process (BP), molecular function (MF) and cellular component (CC) categories. **d** Bar plot of the significant KEGG pathways enriched with DEARGs. *Notes* In plots **a**–**d**, only the top 20 (most significant) terms in each cluster are shown

To evaluate the biological function of these 153 DEARGs, we performed GO and pathway enrichment analyses. The GO analysis results showed that DEARGs were significantly enriched in the biological process (BP) category, including cell death, apoptosis, and autophagy (Fig. 3a). For the molecular function (MF) category, the DEARGs were enriched in autophagic vacuoles, cytosol, and cell fraction (Fig. 3b). In addition, the GO cellular component (CC) category analysis showed that the DEARGs were significantly enriched in protein dimerization activity, protein heterodimerization activity, and cysteine-type peptidase activity (Fig. 3c). From the KEGG pathway enrichment analysis results, we found that the DEARGs were enriched in pathways in cancer, regulation of autophagy, mTOR signaling pathway, NOD-like receptor signaling pathway, and Toll-like receptor signaling pathway, mainly related to cancer and immunity (Fig. 3d).

### PPI network construction and module selection

**Fig. 4:**
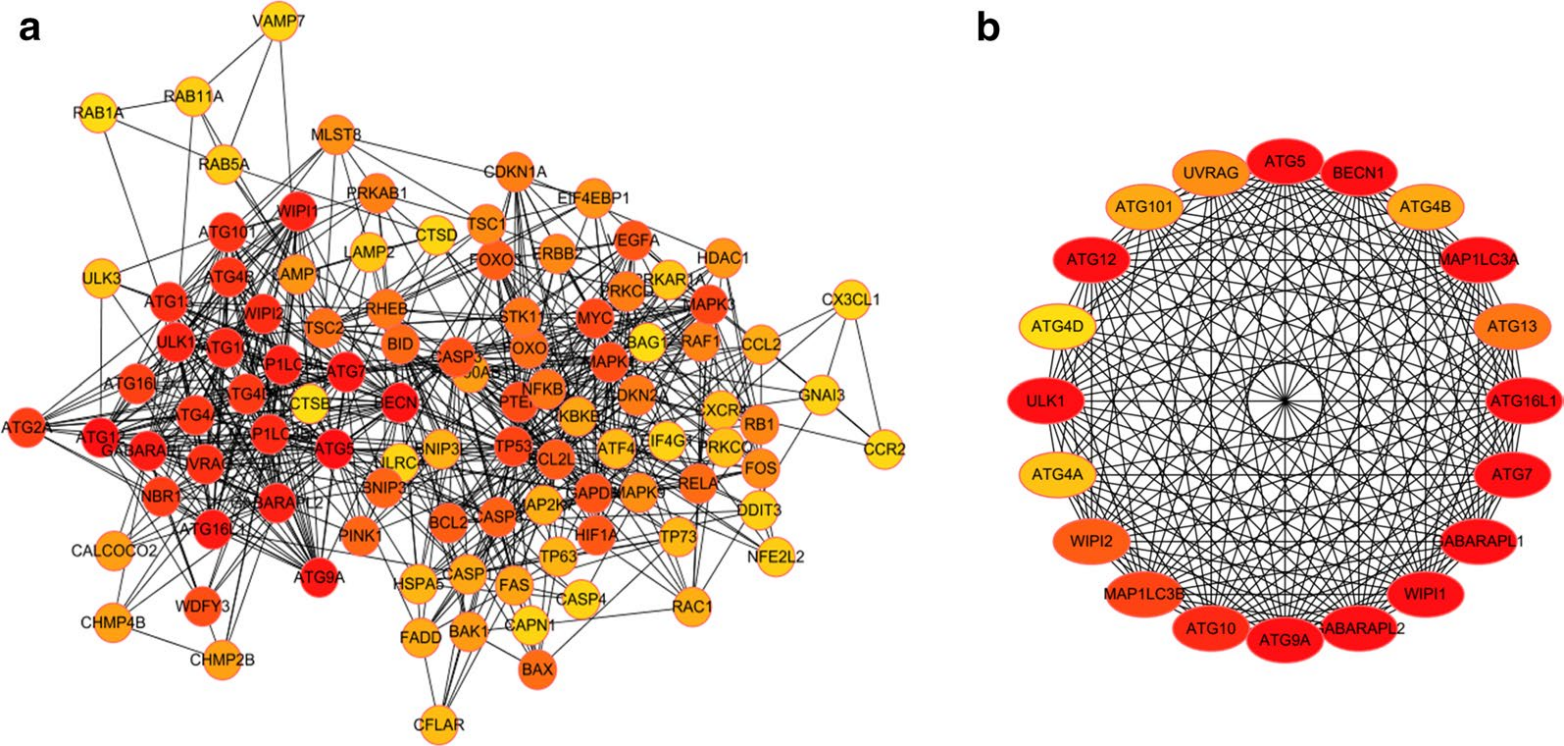
Protein–protein interaction (PPI) network and the most significant modules. **a** The PPI network constructed using the STRING database for DEARGs. **b** The most significant modules obtained from the PPI network with preset criteria

**Fig. 5:**
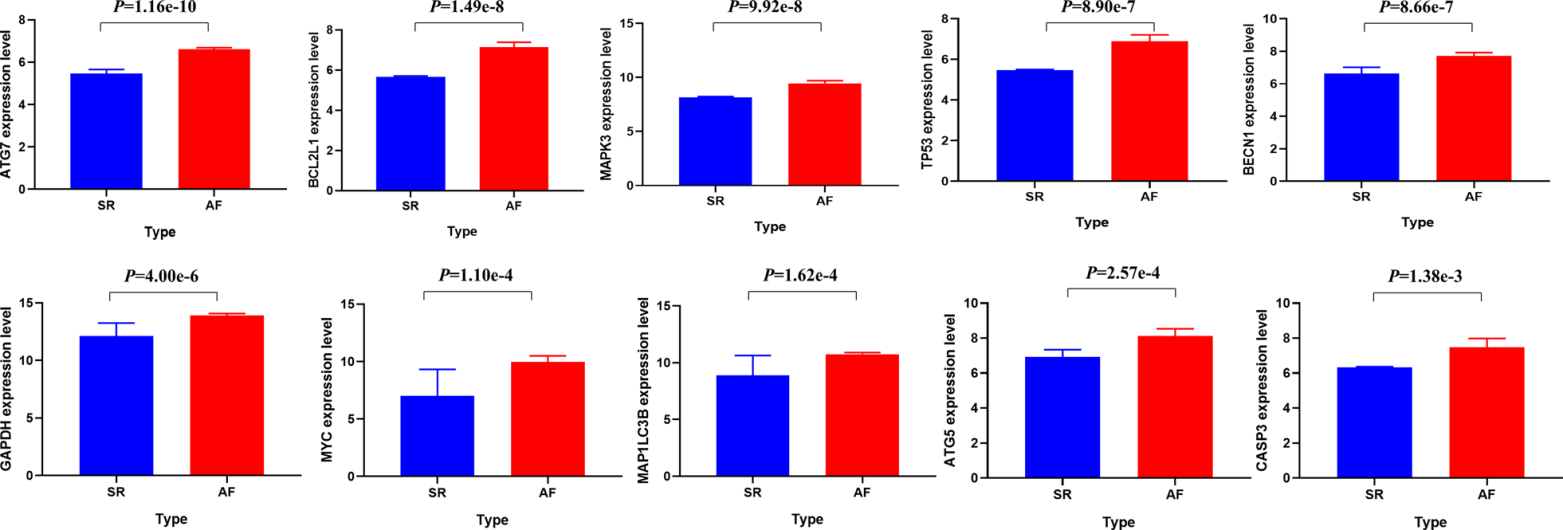
The significant differences in the expression levels of 10 DEARGs between valvular heart disease (VHD) patients with atrial fibrillation (AF) and individuals with normal sinus rhythm (SR) are illustrated

Next, we constructed a PPI network of these 153 DEARGs to identify the key modules and hub genes in valvular AF (Fig. 4a). The five most significant modules were then revealed using the MCODE plugin with the preset cutoff criteria (Fig. 4b). Moreover, we screened the top ten hub nodes with the highest degrees in the PPI network. Ten hub nodes, BECN1, CASP3, GAPDH, TP53, ATG5, ATG7, MAPK3, BCL2L1, MYC, and MAP1LC3B, were considered hub genes related to AF genesis and progression (Table 1). Furthermore, we found that the expression levels of the ten hub genes were up-regulated in the AF-VHD group (*P* < 0.01) (Fig. 5).

**Table 1:** Top ten hub nodes with the highest degrees in the protein–protein interaction (PPI) network

Gene names	Degree	logFC	AveExpr	t	*P*-Value	adj. *P*-Val	B
BECN1	86	1.09	7.55	7.48	3.04E−07	7.70E−07	6.58
CASP3	86	1.15	7.29	4.07	5.88E−04	7.35E−04	− 1.07
GAPDH	82	1.79	13.61	7.11	6.47E−07	1.48E−06	5.81
TP53	81	1.42	6.67	7.72	1.86E−07	5.06E−07	7.08
ATG5	72	1.20	7.93	4.86	9.16E−05	1.28E−04	0.79
ATG7	71	1.15	6.43	11.89	1.41E−10	2.09E−09	14.39
MAPK3	65	1.30	9.25	8.79	2.44E−08	9.49E−08	9.15
BCL2L1	65	1.46	6.90	10.00	2.87E−09	1.77E−08	11.32
MYC	64	2.95	9.50	5.42	2.56E−05	3.96E−05	2.07
MAP1LC3B	63	1.86	10.45	5.18	4.45E−05	6.57E−05	1.52

### Construction of a TF/mRNA/miRNA regulatory network in valvular AF

**Fig. 6:**
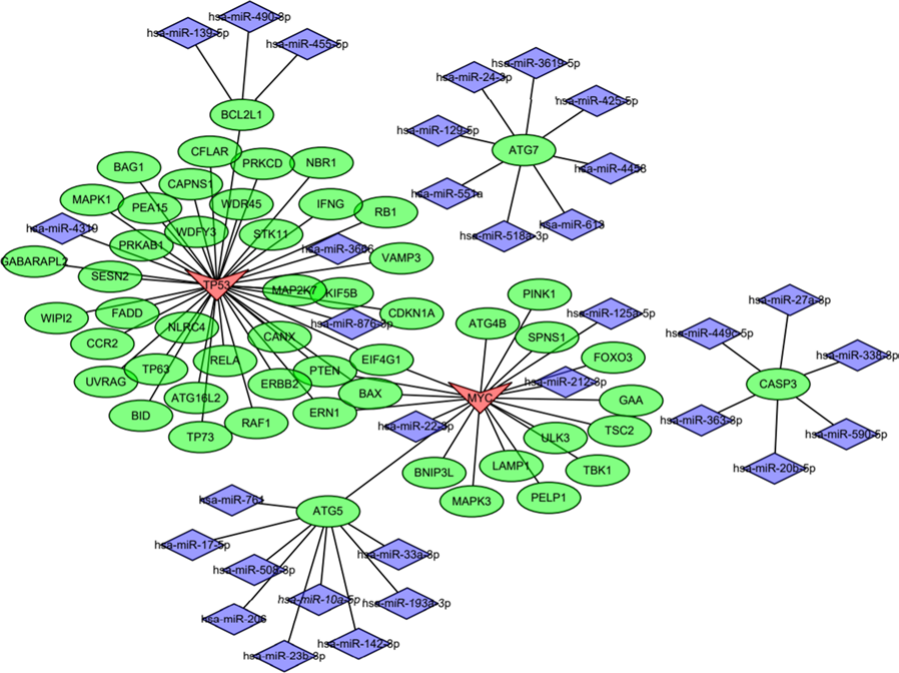
View of the transcription factor (TF)/mRNA/miRNA regulatory network. TFs, mRNAs, and miRNAs are represented by v-shaped frames, ellipses, and diamonds, respectively

We then investigated the regulatory mechanism of the ten hub genes in valvular AF by constructing an autophagy-related TF/mRNA/miRNA network. Using the miRNA-DEARG and TF-DEARG interactions obtained from the Enrichr database, we found that the network consisted of two TFs, nine targeted mRNAs, and 35 miRNAs (Fig. 6). Specifically, most of the DEARGs were regulated by two TFs (MYC and TP53).

### Immune cell infiltration analysis

**Fig. 7:**
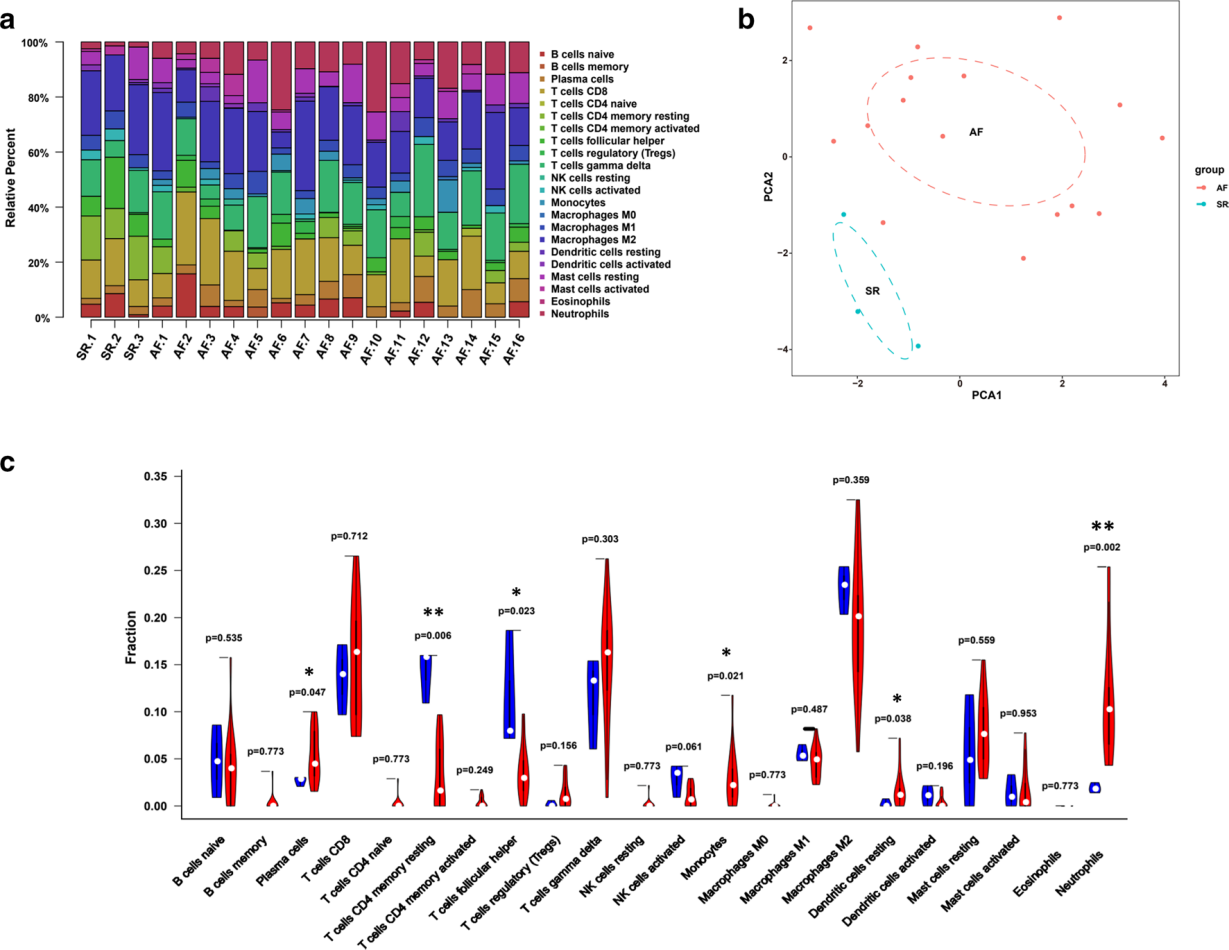
The immune phenotype landscape in the VHD samples with AF and SR. **a** Bar plot showing the relative proportions of 22 immune cell populations in the VHD tissues. **b** Principal component analysis (PCA) performed on all VHD samples. The first two PCs, which accounted for the majority of the model variations between groups, are shown. **c** Violin plot comparing immune cell compositions in the VHD patients with AF and individuals with normal SR. (The AF-VHD group is depicted in red, and the control group is depicted in blue. *P* < 0.05 was considered statistically significant)

To reveal the potential mechanisms of the enriched immune pathways, we conducted an immune cell infiltration analysis with VHD tissues. The histogram map of the relative composition of the 22 immune cell types in the VHD showed that T cells CD8, T cells gamma delta, and macrophages M2 were the most abundant immune cell subpopulations (Fig. 7a). By principal component analysis (PCA), the fractions of the immune cells from AF patient samples and SR controls demonstrated distinct intergroup bias and significant individual differences (Fig. 7b). Using the CIBERSORT algorithm, the results of the Wilcoxon rank-sum test suggested that the fractions of the T cells CD4 memory resting (*P* = 0.006) and T follicular helper cells (*P* = 0.023) in the VHD with AF samples were relatively smaller than those in the VHD with SR samples, and the fractions of plasma cells (*P* = 0.047), monocytes (*P* = 0.021), dendritic cells resting (*P* = 0.038), and neutrophils (*P* = 0.002) were relatively larger in the VHD with AF samples (Fig. 7c). The six differentially infiltrated immune cells were then included in co-expression analysis.

### Co-expression analysis of the DEARGs and AF-VHD related immune cells

**Fig. 8:**
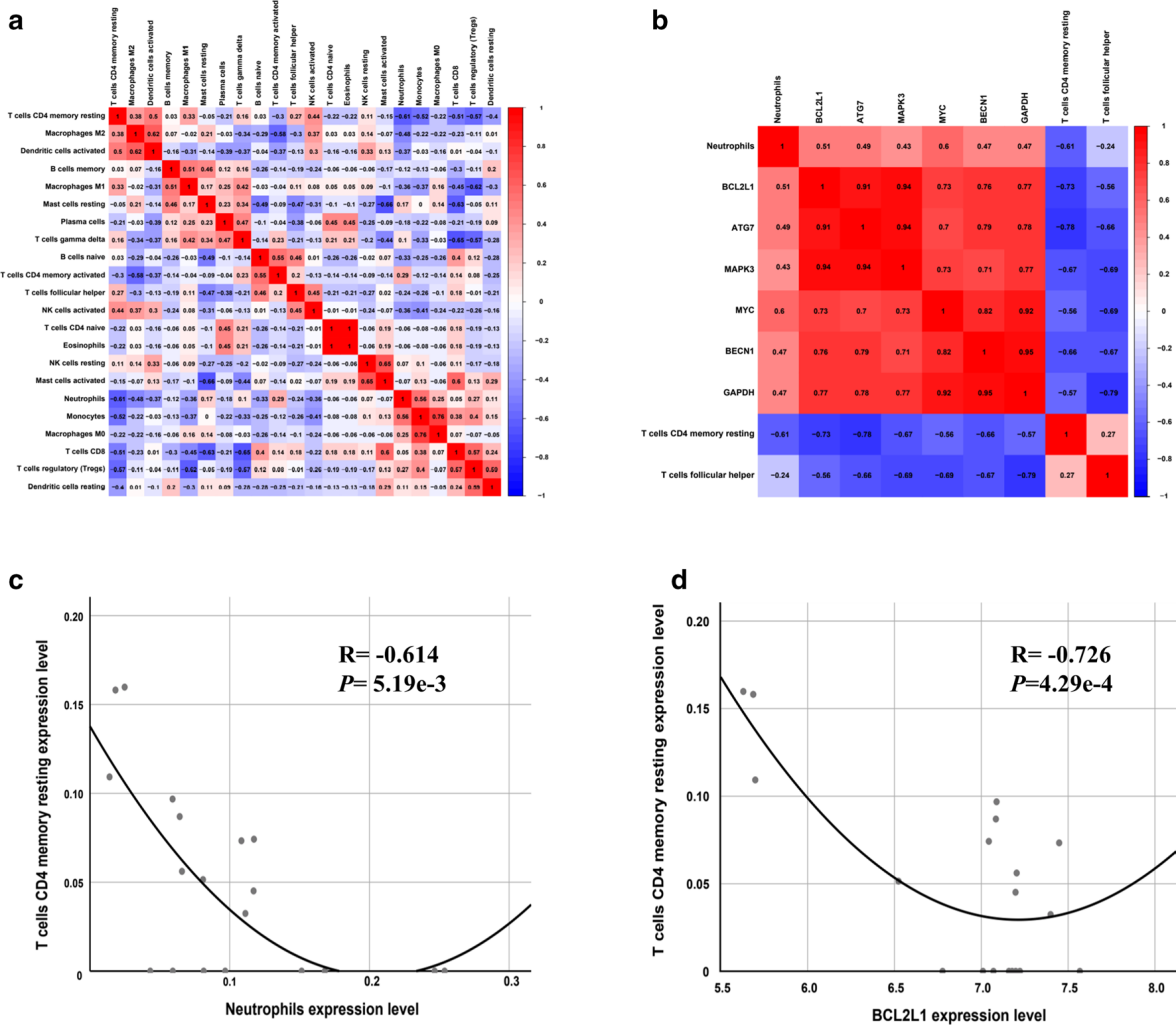
Co-expression patterns of the DEARGs and immune cell subpopulations. **a** Pearson correlation analysis of different infiltrating immune cell subpopulations and **b** the relationships between the DEARGs and infiltrating immune cells in the VHD tissues. **c** Scatterplots further delineate the exact association between neutrophils and T cells CD4 memory resting (*P* = 5.19e**−**3, R =  − 0.614) and **d** the relationship between BCL2L1 and T cells CD4 memory resting (*P* = 4.29e**−**4, R =  − 0.726)

The underlying communicative mechanisms of DEARGs and immune cells were explored by Pearson correlation analysis. First, we explored potential correlations between 22 different immune cell types (Fig. 8a). The resulting heatmap showed that the percentages of different subpopulations of infiltrating immune cells showed weak to moderate correlations. As shown in Fig. 8c, neutrophils and T cells CD4 memory resting (*P* = 5.19e−3, R =  − 0.614) showed a correlation.

Subsequently, the correlation between the DEARGs and immune cells was further analyzed. The results revealed that the expression levels of certain DEARGs, including BECN1, GAPDH, ATG7, MAPK3, BCL2L1, and MYC, had significant associations with the infiltration levels of T cells CD4 memory resting, T cells follicular helper, and neutrophils in the AF-VHD samples (Table 2). The correlation between the DEARGs and significant immune cell subpopulations is illustrated in Fig. 8b, and BCL2L1 and T cells CD4 memory resting (*P* = 4.29e−4, R =  − 0.726) showed a good correlation (Fig. 8d). These findings strongly suggest that DEARGs, including BECN1, GAPDH, ATG7, MAPK3 BCL2L1, and MYC, play specific regulatory roles in immune infiltration cells, especially T cells CD4 memory resting, T cells follicular helper, and neutrophils.

**Table 2:** Correlation analysis between differentially expressed autophagy-related genes (DEARGs) and immune cell subpopulations

Gene names	Plasma cells		T cells CD4 memory resting		T cells follicular helper		Monocytes		Dendritic cells resting		Neutrophils	
	R	*P*	R	*P*	R	*P*	R	*P*	R	*P*	R	*P*
BECN1	0.3	0.21	− 0.066	**	− 0.67	**	0.31	0.19	0.35	0.14	0.47	*
CASP3	0.41	0.08	− 0.57	*	− 0.35	0.14	− 0.04	0.87	0.19	0.44	0.48	*
GAPDH	0.24	0.31	− 0.57	*	− 0.8	***	0.37	0.11	0.3	0.22	0.47	*
TP53	0.45	0.05	− 0.65	**	− 0.61	**	0.35	0.14	0.18	0.47	0.3	0.21
ATG5	0.42	0.08	− 0.56	*	− 0.49	*	− 0.16	0.52	0.27	0.26	0.36	0.13
ATG7	0.37	0.12	− 0.78	***	− 0.65	**	0.43	0.07	0.18	0.46	0.49	*
MAPK3	0.31	0.19	− 0.67	**	− 0.69	**	0.49	*	0.2	0.41	0.43	0.07
BCL2L1	0.33	0.17	− 0.73	***	− 0.56	*	0.5	*	0.2	0.42	0.51	*
MYC	0.11	0.67	− 0.56	*	− 0.69	**	0.43	0.07	0.26	0.28	0.6	**
MAP1LC3B	0.28	0.24	− 0.48	*	− 0.77	***	0.22	0.37	0.22	0.36	0.43	0.07

*P < 0.05; **P < 0.01; ***P < 0.001

## Discussion

In the present study, we applied network analysis and the CIBERSROT algorithm to identify biologically significant DEARGs and immune cells related to AF genesis. We revealed the key correlated regulators of six DEARGs (BECN1, GAPDH, ATG7, MAPK3, BCL2L1, and MYC) and three immune cell subpopulations (T cells CD4 memory resting, T cells follicular helper, and neutrophils) that were unique to patients who developed AF after VHD.

Through meta-analysis of associated genome-wide association studies, Ellinor et al. identified six AF susceptibility loci (PRRX1, CAV1, SYNE2, FBP1/2, HCN4, and SYNPO2L-MYOZ1) involved in cardiac electrical and structural remodeling [22]. In addition, performing a bioinformatics analysis, Zou et al. found four co-expressed genes (ZNF566, PDZK1IP1, ZFHX3, and PITX2) significantly associated with AF-related stroke [23]. In this study, we identified six autophagy-related genes (BECN1, GAPDH [24], ATG7, MAPK3 [25], BCL2L1, and MYC [26, 27]) associated with AF genesis. Currently, numerous studies have revealed that cardiovascular diseases are associated with autophagic genes, both positively [28] and negatively [29]. For example, a recent study demonstrated that FAK-mediated phosphorylation of BECN1 negatively regulated cardiomyocyte autophagy, thereby initiating hypertrophic cardiac growth [30]. Down-regulation of GAPDH reduced H9C2 cardiomyoblast death following acute hypoxia and reoxygenation injury [31]. A study by Yuan et al. [32] showed that ATG7 expression was up-regulated in the atria of AF patients and rabbit models of rapid atrial pacing. In addition, lentivirus-mediated ATG7-knockdown in rabbits was found to protect against atrial electrical remodeling in intracardiac experiments [32]. In an acute myocardial infarction rat model, miRNA-15b was demonstrated to deteriorate cardiomyocyte apoptosis by post-transcriptionally down-regulating the expression of BCL-2 and MAPK3 [33]. High-dose administration of chlorpromazine led to an elevated expression level of BCL2L1 and various cardiovascular disorders, such as arrhythmia and myocardial fibrosis [34]. The up-regulation of c-MYC has been shown to be a central component in the Wnt/β-catenin/c-MYC axis mediated cardiac remodeling abnormalities in heart failure [35].

Currently, pharmacologic therapy of AF is based on anti-arrhythmic drugs, and the interventional treatment is mostly radiofrequency catheter ablation [36]. Other effective preventive medications are needed to curb the occurrence of AF, and some drugs have been reported to induce apoptotic effects in various diseases targeting these identified DEARGs. For instance, Wei et al. described apogossypol derivatives that inhibit antiapoptotic Bcl-2 family proteins [37]. Meanwhile, Germann et al. showed that ulixertinib, targeting the MAPK3 signaling pathway, reduced the proliferation and enhanced the caspase activity of sensitive cancer cells [38].

To comprehensively investigate the biological function of the DEARGs in valvular AF, we performed functional enrichment analysis. The results showed that autophagy genes were significantly enriched in autophagic and inflammatory signaling pathways. These results were consistent with a previous study, which demonstrated that the DEGs identified between the AF-VHD and SR-VHD groups were primarily associated with inflammatory responses [23]. The function of TFs and miRNAs is to regulate gene expression, which is closely involved in the genesis and progression of valvular AF. In our study, the TF/mRNA/miRNA network analysis revealed that most hub genes were associated with two TFs (MYC and TP53), and 35 miRNAs mainly targeted 3 DEARGs (ATG7, BCL2L2 and MYC). We hypothesized that TFs and miRNAs might be critical for AF development by regulating hub gene expression. Additional studies are needed to explore the specific mechanism of TFs and miRNAs in valvular AF.

Previous studies have reported that autophagy and immune infiltration are closely linked to the development and progression of AF [9, 39, 40]. However, no systematic investigation or research has been conducted to elucidate the communicative functions of autophagy and immune infiltration in VHD patients who develop AF. Thus, we comprehensively analyzed the potential mechanisms of DEARGs and immune infiltration cells in AF-VHD, which has rarely been the foci of prior studies.

We also found that three immune cell subpopulations (T cells CD4 memory resting, T follicular helper cells, and neutrophils) were related to AF genesis in VHD patients. Previous studies reported that neutrophils constitute the majority of the inflammatory cells in AF patients undergoing pericardiotomy, atriotomy, or catheter ablation [41, 42]. Moreover, an elevated neutrophil–lymphocyte ratio in postoperative AF patients was highly consistent with the correlation results in our study. The level of neutrophils was inversely correlated with that of CD4 T cells. In addition, CD4 T cells (T cells CD4 memory resting and T follicular helper cells) have different roles during chronic inflammation, and their activation might be mediated through interactions with Toll-like receptor 2 (TLR2) and TLR4 [40, 43]. However, little is known about the specific mechanism of CD4 T cells in the pathogenesis of AF. To confirm our conclusions, experimental mechanistic research should be carried out both in vitro and in vivo in future studies.

The role of autophagy can be seen in a range of cell types involved in immunity, such as lymphocytes, dendritic cells (DCs) and myeloid cells, which contribute to inflammatory responses in diverse pathophysiological processes [14]. BECN1 knockdown in mesenchymal stem cells lead to autophagy suppression, inducing inhibitory effects on T lymphocyte infiltration [44]. In mice and humans, an immunomodulatory drug, dimethyl fumarate, inactivates the catalytic cysteine of GAPDH, which activates myeloid and lymphoid cells [45]. IL-17A was reported to positively impact microglial autophagy and inflammation by promoting the essential autophagy gene ATG7 [46]. Compared to the DCs of MAPK3(^+^/^+^) mice, the DCs of MAPK3 (^−^/^−^) mice possessed a superior capacity to activate and prime naïve T cells into a functional phenotype [47]. Autophagy also plays an essential role in maintaining Treg cells. Inhibition of c-MYC following autophagy deficiency causes Treg cell apoptosis and lineage instability [48]. In summary, we inferred that DEARGs might have significant roles in the occurrence of AF by regulating innate and adaptive immunity through these immune cells. More research directly investigating molecular mechanisms is required to validate the communication between those DEARGs and immune cells.

## Conclusions

We constructed an autophagy-related TF/mRNA/miRNA network and performed immune infiltration analysis to propose novel regulatory mechanisms for valvular AF occurrence. The DEARGs (BECN1, GAPDH, ATG7, MAPK3, BCL2L1, and MYC) and immune cells (T cells CD4 memory resting, T cells follicular helper, and neutrophils) identified in our study may be critical in AF genesis and provide potential predictive and therapeutic strategies for AF patients. The present study not only increases the understanding of the regulatory mechanism of DEARGs and immune cells in valvular AF but also provides candidates for potential diagnostic biomarkers or therapeutic targets in VHD patients developing AF.

## Supplementary Information

**Additional file 1.** Supplemental methods.

## Data Availability

The data used to support the findings of this study are available from the corresponding author upon request. In addition, we thank the contributors of the GEO databases for the availability of the data. The gene expression profiles in the GSE41177 dataset were downloaded from the Gene Expression Omnibus (GEO) database (https://www.ncbi.nlm.nih.gov/geo/query/acc.cgi?acc=GSE41177).

## Abbreviations

AF: Atrial fibrillation
VHD: Valvular heart disease
SR: Sinus rhythm
PPI: Protein–protein interaction
DEARGs: Differentially expressed autophagy-related genes
DEmRNAs: Differentially expressed mRNAs
FC: Fold-change
HADb: Human Autophagy Database
KEGG: Kyoto Encyclopedia of Genes and Genomes
CC: Cellular component
MF: Molecular function
BP: Biological process
STRING: Search Tool for the Retrieval of Interacting Genes
TFs: Transcription factors
CIBERSORT: Cell Type Identification by Estimating Relative Subsets of RNA Transcripts Algorithm
PCA: Principal component analysis
DCs: Dendritic cells
