# Supplementary material for: Comprehensive analysis of autophagy-related genes and patterns of immune cell infiltration in valvular atrial fibrillation
Source: BMC Cardiovasc Disord. 2021 Mar 11;21:132. doi: 10.1186/s12872-021-01939-1 (PMC7948357; doi:10.1186/s12872-021-01939-1)
Supplement: Supplementary file 1 — Additional file 1. Supplemental methods. [file 12872_2021_1939_MOESM1_ESM.docx]

**Comprehensive analysis of** **autophagy-related genes and patterns of immune cell infiltration in valvular atrial fibrillation**

Ao Liu^1^; Kangni Jia^1^; Huaibin Liang^2^; Qi Jin^1*^

^1^Department of Cardiology, Shanghai Ruijin Hospital, Shanghai Jiao Tong University School of Medicine, Shanghai, China

^2^Department of Neurology, Shanghai Ruijin Hospital, Shanghai Jiao Tong University School of Medicine, Shanghai, China

***Corresponding author**: Qi Jin, MD, PhD

Fax number: +86-021-64310871

Telephone number: +86-021-64370045-673333

E-mail: jinqi127@163.com

Address: Department of Cardiology, Shanghai Ruijin Hospital, No. 197, Shanghai Ruijin Er Road, Shanghai, China, 200025

**Supplementary methods.**

The raw gene expression data were assayed using RMA algorithm and log2 transformed perfect match. We applied the Benjamini-Hochberg procedure to adjust original *P*-values and the FDR method to calculate fold-changes. Besides, R package of “limma” (*Nucleic Acids Res* 2015. 43(7):e47) was implemented to assess differentially expressed mRNAs.

For the identified differentially expressed autophagy-related genes (DEARGs), we generated heatmaps using the ggplot and heatmap packages in the R platform. DAVID 6.8 (http://david.ncifcrf.gov/tools.jsp) is a set of functional annotation tools used to analyze the biological relevance behind massive genes. *P* < 0.05 was considered statistically significant. Gene Ontology (GO) analysis includes three ontologies, cellular component (CC), molecular function (MF), and biological process (BP).

Molecular complex detection (MCODE), a plugin in Cytoscape, was used to identify the most significant modules in the protein-protein interaction (PPI) network. The conditions for selection were: MCODE scores > 5, degree cutoff = 2, node score cutoff = 0.2, K-core = 2, and max depth = 100.

Correlations between firm associated DEARGs and immune cells were explored via correlation modules. The expression scatterplots between DEARGs and immune cells were further generated through correlation modules, together with the estimated Pearson correlation coefficient and statistical significance. DEARGs or immune cells were represented on the x-axis with gene symbols or immune cell names, and related immune cells appeared on the y-axis as immune cell names.
